# Persistent Monitoring for Points of Interest with Different Data Update Deadlines

**DOI:** 10.3390/s24041224

**Published:** 2024-02-14

**Authors:** Qing Guo, Jian Peng

**Affiliations:** College of Computer Science, Sichuan University, Chengdu 610065, China; guoqing1@stu.scu.edu.cn

**Keywords:** Points of Interest, data collection, flying tour plan, approximation algorithm

## Abstract

In this paper, we study the regular sensory data collection of Points of Interest (PoIs) with multiple Unmanned Aerial Vehicles (UAVs) during an extended monitoring period, where each PoI is visited multiple times before its data update deadline to keep the data fresh. We observe that most existing studies ignored the important differences in the data stored in the PoIs, scheduled a plan that dispatched UAVs to visit all PoIs before the same deadline, and simply repeated the plan during the monitoring period, which undoubtedly increased the service cost of the UAVs. Considering the specific data update deadline of each PoI, we formulate a novel UAV cost minimization problem to collect the data stored in each PoI before its deadline by finding a series of plans for UAVs such that the service cost of the UAVs during the monitoring period is minimized; the service cost of the UAVs is composed of the consumed energy of the UAVs utilized for hovering for data collection and the consumed energy of the UAVs utilized for flying. To deal with the above NP-hard problem, we devise an approximation algorithm by grouping the PoIs and accessing them in batches. Then, we analyze the proposed algorithm and evaluate the performance of the algorithm through experimental simulations. The experimental results show that the proposed algorithm is very promising.

## 1. Introduction

With the development of flight control and navigation, material engineering, computational science, and information and communication technology in recent years, UAVs have the advantages of cheap cost, convenient operation, and high expandability. UAVs have gradually evolved into integrated, unmanned systems that include flight platforms, image acquisitions, mission payloads, data links, and other multi-functional modules [1,2,3]. Owing to the advantages of swift mobility, high cost-effectiveness, high flexibility, and strong expandability, UAVs have now been widely employed in scenarios such as geographic surveying and mapping, material delivery, network services, patrol, and monitoring [4,5,6,7]. In particular, UAVs equipped with digital cameras, thermal infrared imagers, and Internet of Things data collectors can be dispatched to efficiently collect data from PoIs in monitoring areas without the need for telecommunication infrastructure, e.g., cell towers and cables, and without being restricted by environmental factors, e.g., roads and geography [8,9,10]. As a result, UAVs are widely used in various monitoring applications. In disaster areas, UAVs are emerging as a promising method for collecting critical information about the areas by taking photos and videos for PoIs (e.g., collapsed buildings, schools, and malls) with their onboard lightweight cameras and/or thermal infrared imagers or by collecting data from Internet of Things (IoT) devices (i.e., sensors) located in PoIs with their onboard IoT data collectors. For example, UAVs equipped with digital cameras were dispatched for conducting damage assessment in the wake of the natural disasters Hurricanes Florence and Michael in 2018 [8]. Another good example is the long-term environmental monitoring of climate change by using multiple UAVs hovering over a vast area of interest over an extended time period [9].

How to schedule UAVs to perform efficient data collection work has received more and more attention and has become a cutting-edge research problem. In recent years, many studies have taken into account the employment of UAVs to collect data from PoIs or sensors under data collection delay limits [11,12,13,14,15,16]. Liu et al. [13] used a single UAV to collect data from nodes when considering the “age of information” (AoI) of data, that is, the time from the generation of the data to the delivery of the data to the base station. They proposed two measurement standards for data “age of information”, the maximum data freshness and the average data freshness. To maximize the above two standards, they employed the dynamic programming algorithm to recursively obtain the optimal trajectory of the UAV. As the computational complexity of dynamic programming algorithms increases rapidly with the increase in network size, a genetic algorithm was proposed to obtain the approximate optimal UAV trajectory for large-scale networks. Zhang et al. [16] considered certain data collection latency constraints of IoT devices, and they investigated the use of UAVs to collect data from all devices to achieve the goal of minimizing the number of UAVs. Although they consider the data collection latency limit of devices in the network, they set the same data collection latency limit for all devices. The studies [11,12,14] considered that different nodes have different data collection time constraints. Samir et al. [14] employed one UAV to collect data, but they could not meet all the data collection time constraints of nodes as the energy of a UAV is limited. And their goal was to maximize the number of served nodes. Ghdiri et al. [12] investigated how to minimize the number of UAVs required and the consumed energy of the UAVs, but they only considered one round of data collection and did not consider the case of repeated data collection over a long monitoring period. Drucker et al. [11] investigated the use of multiple UAVs to repeatedly visit targets over a long period, with the goal of minimizing the number of UAVs required.

Unlike most of the above papers, which only considered the scenario of collecting data once, in this paper, we consider a network with PoIs that need to be monitored during a long monitoring period, which means that the PoIs need to be revisited several times over the monitoring period, and there should be a series of data collection plans. In order to ensure the “effectiveness” of the data collected by the UAVs, the data collection delay of a PoI cannot be greater than its given data update deadline, where the data collection delay of the PoI is the time interval between two successive receipts of its data by the base station. Also, we consider that the data update deadline of each PoI is not the same, so it is not necessary to include all PoIs in each data collection plan. Simply including all PoIs in each round of data collection plan would also increase the service cost of UAVs.

There are several challenges that we need to address, as follows. When considering the different data update deadlines of the PoIs, it is necessary to decide the number of data collection plans during the long monitoring period and which PoIs need to be included in each data collection plan. We need to assign data collection tasks to multiple UAVs after determining the set of PoIs to be included in each data collection plan. We need to arrange the flying tours of UAVs in each data collection plan so that the service cost of the UAVs is minimized.

To deal with the above challenges, the main contributions of this paper are summarized as follows.

- We first propose a UAV service cost minimization problem, which aims to find a series of data collection plans for a given number of UAVs over the monitoring period, such that the service cost (the total consumed energy) of the UAVs is minimized over the monitoring period.
- For the NP-hard UAV service cost minimization problem, we propose an approximation algorithm and prove the approximate ratio of the algorithm.
- We finally evaluate the performance of the proposed algorithms via simulation environments, and experimental results show that the proposed algorithms are very promising.In particular, the total UAV consumed energy of the data collection plans delivered by the proposed algorithms is only 35% to 85% of those by the benchmarks.

The rest of this paper is organized as follows. Section 2 introduces the network model and defines the UAV service cost minimization problem. Section 3 presents an algorithm for the problem and analyzes the algorithm. Section 4 evaluates the performance of the proposed algorithm through extensive simulation experiments. Finally, Section 5 concludes this paper.

## 2. Preliminaries

In this section, we first introduce the network model, and then define the problem precisely.

### 2.1. Network Model

**Figure 1:**
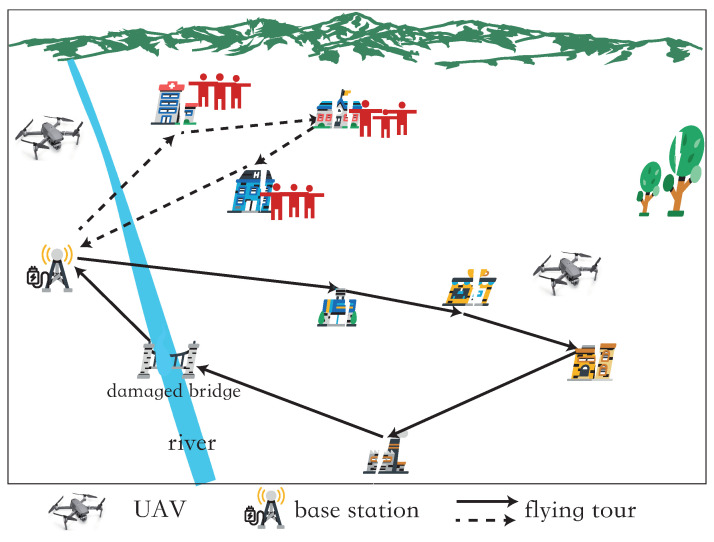
An illustration of the network.

A three-dimensional region to be monitored is considered with *n* PoIs, i.e., $v_{1}$, $v_{2}$, *…*, $v_{n}$. Here, *V* is used to denote the set of the above PoIs, i.e., $V=\{v_{1},v_{2},\ldots ,v_{n}\}$. The coordinates of each PoI $v_{i}$ are $\left(x_{i},y_{i},z_{i}\right)$, where 1≤i≤n and n=|V|. There is a base station, BS, in the network for UAV takeoff, landing, and energy replenishment. All the data collected by UAVs is aggregated and processed at the base station BS. The monitoring period of the network is *T*. In the period *T*, *K* UAVs are dispatched to periodically collect data from all PoIs and ensure that the data collection delay of each PoI $v_{i}$ does not exceed its data update deadline $T_{i}$, where $T_{i}$ is a given constant. And the data collection delay of a PoI is equal to the time period between two consecutive times that the base station receives the data of the PoI (Figure 1).

For each UAV *k*(1≤k≤K), the consumed energy to visit each PoI $v_{i}$ assigned to it is divided into two components, the hovering energy $\varpi_{1}\left(v_{i}\right)$ required to collect data from $v_{i}$ and the flying energy $\varpi_{2}\left(v_{i-1},v_{i}\right)$ required to fly from the last visited PoI $v_{i-1}$ to $v_{i}$. For $\varpi_{1}\left(v_{i}\right)$,

(1) $$\varpi_{1}\left(v_{i}\right)=\xi_{1}\cdot h\left(v_{i}\right),$$

where $\xi_{1}$ is the energy consumption rate of the UAVs when hovering [17,18], $h(v_{i})$ is the hovering time required for the UAVs to collect data from PoI $v_{i}$ [8]. For $\varpi_{2}\left(v_{i-1},v_{i}\right)$,

(2) $$\varpi_{2}\left(v_{i-1},v_{i}\right)=\xi_{2}\cdot f\left(v_{i-1},v_{i}\right)=\xi_{2}\cdot \frac{d(v_{i-1},v_{i})}{\eta },$$

$\xi_{2}$ is the energy consumption rate of the UAVs when flying [17,18], $f(v_{i-1},v_{i})$ is the flying time of the UAVs from PoI $v_{i-1}$ to $v_{i}$, $d(v_{i-1},v_{i})$ is the Euclidean distance of the UAVs from PoI $v_{i-1}$ to $v_{i}$, and η is the flying speed of the UAVs. In fact, there are other factors that affect the consumed energy of the UAVs as well, e.g., data transmission, which are ignored as the consumed energy of the other factors is sufficiently small [10,17].

Assume each UAV k(1≤k≤K) is assigned a set $V_{j}^{k}$ of PoIs at a moment $t_{j}\;\left(0\leq t_{j}\leq T\right)$, and the flying tour of the UAV is assigned as $C_{j}^{k}=BS\to v_{1}\to v_{2}\to \cdots \to v_{n_{k}}\to BS$. It is worth noting that since each UAV departs from the base station BS and returns to BS after collecting data from each PoI, $C_{j}^{k}$ must be a closed tour which contains BS. For each UAV *k*, the total consumed energy $\varpi (C_{j}^{k})$ of the tour $C_{j}^{k}$ that collects data from all PoIs in the subset $V_{j}^{k}$ is the sum of the energy consumed by the UAV to collect data from each PoI $v_{i}$ in $V_{j}^{k}$ and the energy consumed by the UAV to fly between the PoIs, i.e.,

(3) $$\varpi \left(C_{j}^{k}\right)=\sum_{i=1}^{n_{k}}\varpi_{1}\left(v_{i}\right)+\sum_{i=1}^{n_{k}-1}\varpi_{2}\left(v_{i},v_{i+1}\right)+\varpi_{2}\left(BS,v_{1}\right)+\varpi_{2}\left(v_{n_{k}},BS\right),$$

where $n_{k}=\left|V_{j}^{k}\right|$. At the moment $t_{j}$, assume that $C_{j}$ is the set of flying tours of the *K* UAVs, i.e., $C_{j}=\{C_{j}^{1},C_{j}^{2},\ldots ,$$C_{j}^{K}\}$. It is worth noting that since each PoI has a data update deadline, the *K* UAVs may not visit all PoIs in set *V* at each moment $t_{j}$. Here, the binary $\left(C_{j},t_{j}\right)$ is used to denote the data collection plan of the *K* UAVs which starts from the moment $t_{j}$. In the plan $\left(C_{j},t_{j}\right)$, each UAV k(1≤k≤K) departs from the base station BS at the moment $t_{j}$, and the UAV *k* visits all the PoIs involved in the tour $C_{j}^{k}$$\left(C_{j}^{k}\in C_{j}\right)$ one by one, collects the data of each PoI, and finally returns to the BS. Let $V(C_{j}^{k})$ and $V(C_{j})$ be the sets of PoIs contained in $C_{j}^{k}$ and $C_{j}$, respectively. Since $C_{j}=\{C_{j}^{1},C_{j}^{2},\ldots ,$$C_{j}^{K}\}$, we have $V\left(C_{j}\right)=\cup_{k=1}^{K}V\left(C_{j}^{k}\right)$.

Since each PoI in *V* does not have exactly the same data update deadline, PoIs with shorter data update deadlines should be visited more often during the monitoring period *T*. Here, the service cost of the *K* UAVs is defined as the total consumed energy of all UAVs during the monitoring period *T*.

### 2.2. Problem Definition

In this subsection, we define a new problem, i.e., the UAV service cost minimization problem. Given a set $V=\{v_{1},v_{2},\ldots ,v_{n}\}$ of PoIs, the data update deadline $T_{i}$ of each PoI $v_{i}$$\left(v_{i}\in V\right)$, *K* UAVs, the hovering time $h_{i}$ for the UAVs to collect the data of each PoI $v_{i}$, and the network monitoring period *T*, the UAV service cost minimization problem is defined as finding a number *l* of data collection plans $\left(C_{1},t_{1}\right)$, $\left(C_{2}\right),t_{2}$, *…*, $\left(C_{l},t_{l}\right)$, such that the service cost (total consumed energy) of the *K* UAVs over the monitoring period *T* is minimized.

We formally define the problem as follows:

(4) $$\min\;\;\{\sum_{j=1}^{l}\varpi \left(C_{j}\right)=\sum_{j=1}^{l}\sum_{k=1}^{K}\varpi \left(C_{j}^{k}\right)\},\;$$

where $C_{j}=\left\{C_{j}^{1},C_{j}^{2},\ldots ,C_{j}^{K}\right\}$, $C_{j}^{k}$ is the flying tour of UAV *k*, 1≤k≤K, 1≤j≤l, and $0\leq t_{1}<t_{2}<\cdots <t_{l}<T$.

There are two other constraints on the objective function.

(1) For any PoI $v_{i}$ in the set *V*, assume that $\left(C_{j},t_{j}\right)$ and $\left(C_{j^{\prime }},t_{j^{\prime }}\right)$ are two consecutive data collection plans which both contain $v_{i}$. Here, we also assume that $t_{j^{\prime }}>t_{j}$. Then, the time interval between the above two plans is not greater than the data update deadline $T_{i}$ of $v_{i}$, i.e., $t_{j^{\prime }}-t_{j}\leq T_{i}$.

Note that PoI $v_{i}$ must be included in one of the flying tours of $C_{j}$ and $C_{j^{\prime }}$, respectively, and $\left(C_{j},t_{j}\right)$ and $\left(C_{j^{\prime }},t_{j^{\prime }}\right)$ must be two consecutive data collection plans for $v_{i}$; i.e., there does not exist a data plan $\left(C_{j^{\prime \prime }},t_{j^{\prime \prime }}\right)$ such that $v_{i}$ is included in $C_{j^{\prime \prime }}$ when $t_{j}<t_{j^{\prime \prime }}<t_{j^{\prime }}$.

(2) For any PoI $v_{i}$ in the set *V*, the interval between the time when its data was last collected and the end moment of the monitoring period *T* does not exceed the data update deadline $T_{i}$ of $v_{i}$.

In the UAV service cost minimization problem, there are two factors that need to be determined; one is the number *l* of rounds of data collection and the other is the data collection plan in each round, which includes the PoIs for which data need to be collected and the specific flying tours for those PoIs. If more data collection rounds are scheduled during the monitoring period *T*, the number of PoIs whose data will be collected in each round will be lower, and the service cost of the UAVs in each round will be lower. If relatively fewer data collection rounds are scheduled, then the number of PoIs whose data will be collected in each round will be more, and the service cost of the UAVs in each round will increase. Therefore, it is a great challenge to choose the appropriate number of data collection rounds and data collection plans for each round to minimize the total service cost of the UAVs.

## 3. Algorithm

In this section, an approximate algorithm is proposed to minimize the service cost of the UAVs. In the following, the main idea of the approximate algorithm is first presented, and then the specific details of the algorithm are shown. Finally, the approximation ratio of the proposed algorithm is analyzed.

### 3.1. Basic Idea

Given a monitoring period *T*, a set *V* of PoIs, and the data update deadline $T_{i}$ for each PoI $v_{i}$, if there exists a data collection plan in period *T* such that, under the plan, the time interval between two consecutive times that each PoI $v_{i}$ in *V* is visited by a UAV does not exceed its data update deadline $T_{i}$, then the data collection plan is a feasible solution. And if there exists one of the above data collection plans and the total service cost of *K* UAVs is minimized under the plan, then the data collection plan is an optimal solution to the UAV service cost minimization problem. It is worth noting that a data collection plan for the UAV service cost minimization problem is composed of a series of data collection sub-plans.

The approximation algorithm is divided into three steps. The first step is to construct a UAV consumed energy auxiliary graph $G^{\prime }$ through the given information, such that $G^{\prime }$ is a metric graph. The rationale behind the graph transformation is that we will later show that an optimal solution to the UAV service cost minimization problem in graph $G^{\prime }$ is also an optimal solution to the problem in the original graph *G* (see Lemma 1). And an α-approximation solution to the problem in $G^{\prime }$ returns an α-approximation solution to the problem in *G*, where α is a constant with α≥1. It can be seen that it is easier to solve the problem in the auxiliary graph $G^{\prime }$, since the graph *G* is both edge-weighted and node-weighted, while graph $G^{\prime }$ is only edge-weighted.

The second step is to group the PoIs in the set *V* according to the data update deadline $T_{i}$ of each PoI $v_{i}$. According to the data update deadline $T_{i}$ of each PoI $v_{i}$, we set a data collection delay $T_{i}^{\prime }$ for each $v_{i}$, where $\frac{T_{i}}{2}<T_{i}^{\prime }\leq T_{i}$. The algorithm sets that the PoIs in the same group have a same data collection delay, which is equal to the time period between two consecutive times that the base station receives the data of the PoI. And the maximum data collection delay of the PoIs in *V* is denoted as $T_{n}^{\prime }$. According to the data collection delay $T_{i}^{\prime }$ of each $v_{i}$, we divide the monitoring period *T* into time slices and dispatch the UAVs to visit the PoIs every several time slices to guarantee that the data of each PoI is collected within its data collection deadline.

In the third step, the algorithm first finds the data collection plans within the maximum data collection delay $T_{n}^{\prime }$ to ensure that the data of each PoI $v_{i}$ is collected within its data collection deadline $T_{i}$ during $T_{n}^{\prime }$. Then, the algorithm repeats the data collection sub-plans within the period $T_{n}^{\prime }$ and finally obtains a series of data collection sub-plans within the period *T*.

### 3.2. The Detail of the Algorithm

The first step of the algorithm, i.e., the construction process of the auxiliary graph $G^{\prime }$, is described below. Given the network G=(V∪BS,E), the algorithm constructs a new auxiliary graph $G^{\prime }$ with only edge weights from the graph *G*, where $G^{\prime }=\left(V\cup BS,E^{\prime };\varpi^{\prime }:E^{\prime }\to Z^{\geq 0}\right)$, where

(5) $$\varpi^{\prime }\left(v_{i},v_{j}\right)=\xi_{1}\cdot \frac{h\left(v_{i}\right)+h\left(v_{j}\right)}{2}+\xi_{2}\cdot f\left(v_{i},v_{j}\right),$$

$\xi_{1}$ is the energy consumption rate of the UAVs while hovering, $h(v_{i})$ and $h(v_{j})$ are the times that the UAV needs to hover in order to collect data from $v_{i}$ and $v_{j}$, respectively, $\xi_{2}$ is the energy consumption rate of the UAVs while flying, and $f(v_{i},v_{j})$ is the flying time of the UAVs from $v_{i}$ to $v_{j}$.

**Lemma** **1.** *The optimal values of the UAV service cost minimization problem in G and $G^{\prime }$ are equal.*

**Proof.** ** ** Assume that OPT is the optimal value in *G*, and $l^{*}$ data collection plans $\left(C_{1}^{*},t_{1}\right)$, $\left(C_{2}^{*},t_{2}\right)$, *…*, $\left(C_{l^{*}}^{*},t_{l^{*}}\right)$ form an optimal solution to the problem in *G*, where

(6) $$\begin{array}{c} OPT=\sum_{j=1}^{l^{*}}\varpi \left(C_{j}^{*}\right)=\sum_{j=1}^{l^{*}}\sum_{k=1}^{K}\varpi \left(C_{j}^{k}\right). \end{array}$$

Let $C_{j}^{k}=v_{1}\to v_{2}\to \cdots \to v_{q_{k}}\to v_{1}$, where 1≤k≤K and $q_{k}$ is the number of nodes in tour $C_{j}^{k}$. By the definition of the weight $\varpi (C_{j}^{k})$ of $C_{j}^{k}$, we have

(7) $$\begin{array}{ccc} \varpi (C_{j}^{k}) & = & \xi_{1}\cdot \sum_{i=1}^{q_{k}}h(v_{i})+\xi_{2}\cdot \sum_{i=1}^{q_{k}-1}f(v_{i},v_{i+1})+\xi_{2}\cdot f\left(v_{q_{k}},v_{1}\right). \end{array}$$

Similarly, let $l^{\prime }$ data collection plans $\left(\tilde{C_{1}},t_{1^{\prime }}\right)$, $\left(\tilde{C_{2}},t_{2^{\prime }}\right)$, *…*, $\left(\tilde{C_{l^{\prime }}},t_{l^{\prime }}\right)$ form an optimal solution to the problem in graph $G^{\prime }$. Also, let ${OPT}^{\prime }$ be the optimal value in $G^{\prime }$. Then, we have

(8) $$\begin{array}{c} OPT^{\prime }=\sum_{j=1}^{l^{\prime }}\varpi^{\prime }\left(\tilde{C_{k}}\right)=\sum_{j=1}^{l^{\prime }}\sum_{k=1}^{K}\varpi^{\prime }\left(\tilde{C_{j}^{k}}\right). \end{array}$$

Assume that $\tilde{C_{j}^{k}}=v_{1^{\prime }}\to v_{2^{\prime }}\to \cdots \to v_{q_{k}^{\prime }}\to v_{1^{\prime }}$, where 1≤k≤K and $q_{k}^{\prime }$ is the number of nodes in tour $\tilde{C_{j}^{k}}$. Following the definition of the weight $\varpi^{\prime }\left(\tilde{C_{j}^{k}}\right)$ of $\tilde{C_{j}^{k}}$, we have

(9) $$\begin{array}{ccc} \varpi^{\prime }\left(\tilde{C_{j}^{k}}\right) & = & \sum_{i=1}^{q_{k}^{\prime }-1}\left(\xi_{2}\cdot f\left(v_{i},v_{i+1}\right)+\xi_{1}\cdot \frac{h\left(v_{i}\right)+h\left(v_{i+1}\right)}{2}\right)+\\ & & \;\xi_{2}\cdot f\left(v_{q_{k}^{\prime }},v_{1}\right)+\xi_{1}\cdot \frac{h\left(v_{q_{k}^{\prime }}\right)+h\left(v_{1}\right)}{2},\\ & = & \sum_{i=1}^{q_{k}^{\prime }-1}\xi_{2}\cdot f\left(v_{i},v_{i+1}\right)+\xi_{2}\cdot f\left(v_{q_{k}^{\prime }},v_{1}\right)+\xi_{1}\cdot \sum_{i=1}^{q_{k}^{\prime }}h(v_{i}). \end{array}$$

In the following, we show that $OPT\geq {OPT}^{\prime }$ and ${OPT}^{\prime }\geq OPT$. Then, $OPT={OPT}^{\prime }$.

We first show that $OPT\geq {OPT}^{\prime }$. Recall that the $l^{*}$ data collection plans $\left(C_{1}^{*},t_{1}\right)$, $\left(C_{2}^{*},t_{2}\right)$, *…*, $\left(C_{l^{*}}^{*},t_{l^{*}}\right)$ form an optimal solution to the problem in *G*. For each tour $C_{j}^{k}$ contained in the above $l^{*}$ data collection plans, we show that the weights of $C_{j}^{k}$ in graphs *G* and $G^{\prime }$ are equal, i.e., $\varpi \left(C_{j}^{k}\right)=\varpi^{\prime }\left(C_{j}^{k}\right)$, since

(10) $$\begin{array}{ccc} \varpi (C_{j}^{k}) & = & \sum_{i=1}^{q_{k}-1}\xi_{2}\cdot f\left(v_{i},v_{i+1}\right)+\xi_{2}\cdot f\left(v_{q_{k}},v_{1}\right)+\xi_{1}\cdot \sum_{i=1}^{q_{k}}h(v_{i}),\;\mathrm{by}\;\mathrm{Equation}\;(7),\\ & = & \xi_{2}\cdot \sum_{i=1}^{q_{k}-1}\left(f\left(v_{i},v_{i+1}\right)+\xi_{1}\cdot \frac{h\left(v_{i}\right)+h\left(v_{i+1}\right)}{2}\right)+\\ & & \;\;\;\xi_{2}\cdot f\left(v_{q_{k}},v_{1}\right)+\xi_{1}\cdot \frac{h\left(v_{q_{k}}\right)+h\left(v_{1}\right)}{2},\\ & = & \varpi^{\prime }\left(C_{j}^{k}\right),\;\mathrm{by}\;\mathrm{Equation}\;(9). \end{array}$$

Then, we have

(11) $$OPT=\sum_{j=1}^{l^{*}}\varpi \left(C_{j}^{*}\right)=\sum_{j=1}^{l^{*}}\sum_{k=1}^{K}\varpi \left(C_{j}^{k}\right)=\sum_{j=1}^{l^{*}}\sum_{k=1}^{K}\varpi^{\prime }\left(C_{j}^{k}\right)\geq OPT^{\prime },$$

as the data collection plans $\left(C_{1}^{*},t_{1}\right)$, $\left(C_{2}^{*},t_{2}\right)$, *…*, $\left(C_{l^{*}}^{*},t_{l^{*}}\right)$ form a feasible solution to the problem in $G^{\prime }$.

Similarly, we have

(12) $$OPT^{\prime }=\sum_{j=1}^{l^{\prime }}\varpi^{\prime }\left(\tilde{C_{j}}\right)=\sum_{j=1}^{l^{\prime }}\sum_{k=1}^{K}\varpi^{\prime }\left(\tilde{C_{j}^{k}}\right)=\sum_{j=1}^{l^{\prime }}\sum_{k=1}^{K}\varpi \left(\tilde{C_{j}^{k}}\right)\geq OPT,$$

as the data collection plans $\left(\tilde{C_{1}},t_{1^{\prime }}\right)$, $\left(\tilde{C_{2}},t_{2^{\prime }}\right)$, *…*, $\left(\tilde{C_{l^{\prime }}},t_{l^{\prime }}\right)$ also form a feasible solution to the problem in *G*. Following Equations (11) and (12), we have

(13) $$OPT={OPT}^{\prime }.$$

The lemma then follows.    □

The second step of the approximation algorithm, i.e., the grouping process of the set *V* of PoIs, is presented as follows. Firstly, the PoIs in *V* are sorted according to their data update deadlines. Let $T_{1}$, $T_{2}$, *…*, $T_{n}$ be the data update deadlines of $v_{1}$, $v_{2}$, *…*, $v_{n}$, respectively, where $T_{1}\leq T_{2}\leq \cdots \leq T_{n}$. Denote $T_{1}^{\prime }$, $T_{2}^{\prime }$, *…*, $T_{n}^{\prime }$ as the data collection delays of $v_{1}$, $v_{2}$, *…*, $v_{n}$, respectively, where $T_{i}^{\prime }\leq T_{j}^{\prime }$ when $T_{i}\leq T_{j}$. Then, we construct p+1 disjoint time ranges $\left[T_{1},2^{1}T_{1}\right)$, $\left[2^{1}T_{1},2^{2}T_{1}\right)$, *…*, $\left[2^{q}T_{1},2^{q+1}T_{1}\right)$, *…*, $\left[2^{p}T_{1},2^{p+1}T_{1}\right)$, where $p=\lfloor \log_{2}\left(T_{n}/T_{1}\right)\rfloor$. By the definition of the data update deadline of each PoI in set *V*, the data update deadline of any PoI $v_{i}$ must be within the range $\left[T_{1},T_{n}\right]$, i.e., $T_{1}\leq T_{i}\leq T_{n}$. Since the smallest value in the above p+1 time range is $T_{1}$, and the value $2^{p+1}T_{1}=2^{\lfloor \log_{2}\left(T_{n}/T_{1}\right)\rfloor +1}T_{1}<2T_{n}$, the data update deadline $T_{i}$ for each PoI $v_{i}$ must be in the above p+1 time ranges. If the data update deadline $T_{i}$ of $v_{i}$ is in a certain time range $\left[2^{q}T_{1},2^{q+1}T_{1}\right)$(0≤q≤p), i.e., $2^{q}T_{1}\leq T_{i}<2^{q+1}T_{1}$, then $v_{i}$ is classified into the subset $V_{q}$ of *V*. And let $T_{i}^{\prime }=2^{q}T_{1}$ be the data collection delay of $v_{i}$. From the above process, the set *V* can be partitioned into p+1 subsets $V_{0}$, $V_{1}$, *…*, $V_{p}$, where $p=\lfloor \log_{2}\left(T_{n}/T_{1}\right)\rfloor$. From the definition of the subsets, we have that the data collection delays of the PoIs in each subset are the same, i.e., the data collection delays of the PoIs in $V_{0}$, $V_{1}$, *…*, $V_{q}$, *…*, $V_{p}$ are $T_{1}$, $2T_{1}$, *…*, $2^{q}T_{1}$, *…*, $2^{p}T_{1}$, respectively.

From the definition of the data collection delay of each PoI $v_{i}$ in set *V*, we have that the data collection delay $T_{i}^{\prime }$ of $v_{i}$ is no less than half of its data update deadline $T_{i}$, since

(14) $$T_{i}^{\prime }=2^{q}\cdot T_{1}=2^{\left\lfloor \log_{2}\left(T_{i}/T_{1}\right)\right\rfloor }\cdot T_{1}>2^{\log_{2}\left(T_{i}/T_{1}\right)-1}\cdot T_{1}=\frac{T_{i}}{2}.$$

And the data collection delay $T_{i}^{\prime }$ must not be greater than its data update deadline $T_{i}$ because

(15) $$T_{i}^{\prime }=2^{q}\cdot T_{1}=2^{\left\lfloor \log_{2}\left(T_{i}/T_{1}\right)\right\rfloor }\cdot T_{1}\leq 2^{\log_{2}\left(T_{i}/T_{1}\right)}\cdot T_{1}=T_{i}.$$

From the definition of the data collection delay of each PoI, it can be seen that for any two PoIs $v_{i}$ and $v_{j}$ with different data collection delays, if $T_{i}^{\prime }<T_{j}^{\prime }$, then the data collection delay $T_{j}^{\prime }$ of $v_{j}$ must be an integer multiple of the data collection delay $T_{i}^{\prime }$ of $v_{i}$. Among all the PoIs in the set *V*, PoI $v_{n}$ has the largest data collection delay $T_{n}^{\prime }$. Since the monitoring period of the network is *T*, for convenience, set *T* to be an integer multiple of the maximum data collection delay $T_{n}^{\prime }$ and let $T=2mT_{n}^{\prime }=2m2^{p}T_{1}$, where *m* is a positive integer.

In the third step of the algorithm, we first find a series of data collection plans during the maximum data collection delay $T_{n}^{\prime }$ among PoIs. As the length of period *T* is 2m times the length of period $T_{n}^{\prime }$, then we simply repeat the data collection plans during $T_{n}^{\prime }$ 2m times to construct the data collection plans during the monitoring period *T*.

In the following, we describe how to construct the data collection plans during $T_{n}^{\prime }$. As mentioned that the set *V* is partitioned into p+1 sub-sets $V_{0}$, $V_{1}$, *…*, $V_{p}$, where the data collection delay of each PoI in $V_{q}$(0≤q≤p) is $2^{q}T_{1}$, $p=\lfloor \log_{2}\left(T_{n}/T_{1}\right)\rfloor$ and $T_{n}^{\prime }=2^{p}T_{1}$. Firstly, the algorithm divides $T_{n}^{\prime }$, which is equal to $2^{p}T_{1}$, into $2^{p}$ time slices of the same length, each of which is equal to $T_{1}$. Then, it orders the time slices sequentially and determines the set of PoIs to be included in each round of data collection plans based on time slices. Since the PoIs in the sub-set $V_{q}$ have a data collection delay $2^{q}T_{1}$, which means that the PoIs in $V_{0}$ have to be included in the data collection plan for each time slice, whereas the PoIs in the sub-set $V_{1}$ are only included in the data plan for the even-numbered time slice, the PoIs in the sub-set $V_{2}$ are only included in the data plan for the time slice whose serial number is divisible by $2^{2}$, *…*, the PoIs in the sub-set $V_{q}$ are only included in the data plan for the time slice whose serial number is divisible by $2^{q}$, *…*, and the PoIs in the sub-set $V_{p}$ are only included in the data plan for one time slice. Thus, by the algorithm, the data collection plans during the $2^{p}$ time slices are scheduled as follows:

(1) In the first time slice $T_{1}$, dispatch the UAVs to collect data from the PoIs in $V_{0}$. Denote the set of flying tours of the UAVs as $C_{1}$;

(2) In the second time slice $2T_{1}$, dispatch the UAVs to collect data from the PoIs in $V_{0}\cup V_{1}$. And the set of flying tours of the UAVs is noted as $C_{2}$;

(3) In the third time slice $3T_{1}$, dispatch the UAVs to collect data from the PoIs in $V_{0}$. And the set of flying tours of the UAVs is noted as $C_{3}$;

(4) In the fourth time slice $4T_{1}$, the UAVs are scheduled to collect data from the PoIs in $V_{0}\cup V_{1}\cup V_{2}$. And the set of the flying tours of the UAVs is noted as $C_{4}$;

⋮

(*j*) In the *j*-th time slice $jT_{1}$, dispatch the UAVs to collect data from the PoIs in $\cup_{(j\;\mathrm{mod}\;2^{q})=0}V_{q}$. And the set of flying tours of the UAVs is noted as $C_{j}$, where $0\leq q\leq \lfloor \log_{2}j\rfloor$ and $1\leq j\leq 2^{p}$;

⋮

($2^{p}$) In the $2^{p}$th time slice $2^{p}T_{1}$, dispatch the UAVs to collect data from the PoIs in $\cup_{i=1}^{p}V_{i}=V$. And the set of flying tours of the UAVs is noted as $C_{2^{p}}$.

**Figure 2:**
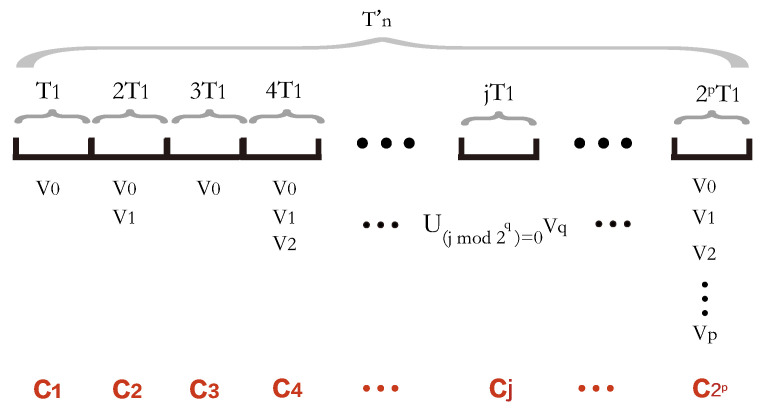
The data collection plans during the $2^{p}$ intervals where the length of each interval is $T_{1}$.

Figure 2 shows the data collection plans during the $2^{p}$ time slices.

As mentioned above, we construct $2^{p}$ data collection plans during the data collection delay $T_{n}^{\prime }$, each of which involves a time slice. In the *j*-th $\left(1\leq j\leq 2^{p}\right)$ time slice, $C_{j}=\left\{C_{j,1},C_{j,2},\ldots ,C_{j,K}\right\}$ is the set of *K* UAV flying tours in the data collection plan during that time slice. The set $C_{j}$ of flying tours, where *K* UAVs collect all the data of PoIs in subset $\cup_{(j\;\mathrm{mod}\;2^{q})=0}V_{q}$, can be obtained by invoking the algorithm for the vehicle routing problem with the objective of minimizing the total cost [19] in graph $G^{\prime }\left[BS\cup \left\{\cup_{(j\;\mathrm{mod}\;2^{q})=0}V_{q}\right\}\right]$. Obviously, the graph $G^{\prime }\left[BS\cup \left\{\cup_{(j\;\mathrm{mod}\;2^{q})=0}V_{q}\right\}\right]$ is a subgraph of the graph $G^{\prime }$.

The PoIs in $V_{0}$ are included in the data collection plans for all of these $2^{p}$ time slices. The PoIs in $V_{1}$ are included in the data collection plans for only $2^{p-1}$ time slices. The PoIs in $V_{0}\cup V_{1}$ are included in the data collection plans for only $2^{p-2}$ time slices.

The PoIs in $V_{0}\cup V_{1}\cup \cdots \cup V_{p}$ are included in the data collection plan for only one time slice. To summarize, only $2^{p-q}$ time slices contain the PoIs in subset $V_{0}\cup V_{1}\cup \cdots \cup V_{q}$, where 0≤q≤p.

Then, during period $\left(0,T_{n}^{\prime }\right)$, we construct $2^{p}$ data collection plans $\left(C_{1},0\right)$, $\left(C_{2},T_{1}\right)$, *…*, $\left(C_{j},\left(j-1\right)T_{1}\right)$, $\left(C_{2^{p}},\left(2^{p}-1\right)T_{1}\right)$, where $\left(C_{j},\left(j-1\right)T_{1}\right)$ denotes that in the *j*-th time slice $jT_{1}$ (i.e., time period $\left(\left(j-1\right)\cdot T_{1},jT_{1}\right)$), *K* UAVs are dispatched to collect the data of PoIs in $\cup_{C_{j,i}\in C_{j}}V\left(C_{j,i}\right)=\cup_{(j\;\mathrm{mod}\;2^{q-1})=0}V_{q}$ following the flying tour set $C_{j}$, where $0\leq q\leq \lfloor \log_{2}j\rfloor$, and $1\leq j\leq 2^{p}$.

Recall that the length of the period *T* is 2m times the length of period $T_{n}^{\prime }$. Finally, we simply repeat the data collection sub-plans within the period $T_{n}^{\prime }$ to obtain a series of data collection sub-plans within the period *T* as follows.

(1) During time slice $T_{n}^{\prime }$ (i.e., time period $\left(0,jT_{1}\right)$), there are $2^{p}$ data collection plans: $\left(C_{1},0\right)$, $\left(C_{2},T_{1}\right)$, *…*, $\left(C_{2^{p}-1},\left(2^{p}-2\right)T_{1}\right)$, $\left(C_{2^{p}},\left(2^{p}-1\right)T_{1}\right)$.

(2) During time slice $2T_{n}^{\prime }$ (i.e., time period $\left(T_{1},2T_{1}\right)$), there are $2^{p}$ data collection plans: $\left(C_{1},T_{n}^{\prime }\right)$, $\left(C_{2},T_{n}^{\prime }+T_{1}\right)$, *…*, $\left(C_{2^{p}-1},T_{n}^{\prime }+\left(2^{p}-2\right)T_{1}\right)$, $\left(C_{2^{p}},T_{n}^{\prime }+\left(2^{p}-1\right)T_{1}\right)$.

⋮

(2m) During time slice $2mT_{n}^{\prime }$ (i.e., time period $\left(\left(2m-1\right)\cdot T_{1},2mT_{1}\right)$), there are $2^{p}$ data collection plans: $\left(C_{1},\left(2m-1\right)T_{n}^{\prime }\right)$, $\left(C_{2},\left(2m-1\right)T_{n}^{\prime }+T_{1}\right)$, *…*, $\left(C_{2^{p}-1},\left(2m-1\right)T_{n}^{\prime }+\left(2^{p}-2\right)T_{1}\right)$, $\left(C_{2^{p}},\left(2m-1\right)T_{n}^{\prime }+\left(2^{p}-1\right)T_{1}\right)$.

**Figure 3:**
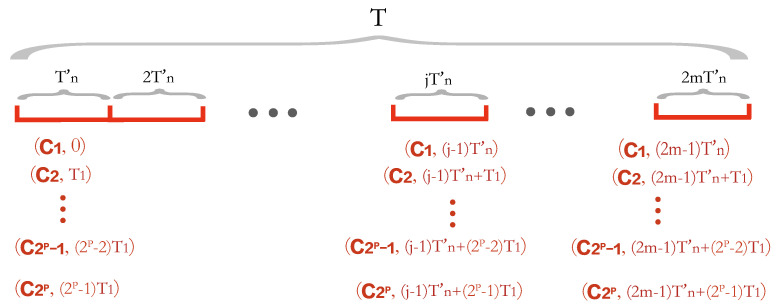
The data collection plans during period *T*.

As shown in Figure 3, there are $2m\cdot 2^{p}$ data collection plans during $T_{n}^{\prime }=2mT_{n}^{\prime }$.

The algorithm for the UAV service cost minimization problem is presented in Algorithm 1 as follows.
**Algorithm 1:** Algorithm for the UAV service cost minimization problem (minCost)**Require:** A network G=(V∪BS,E), the data collection time $h(v_{i})$ of each PoI $v_{i}$ in *V*, the maximum data collection deadline $T_{i}$ of $v_{i}$, and the monitoring period *T*.**Ensure:** A series of data collection plans C in period *T*.1:Construct an auxiliary graph $G^{\prime }=\left(V\cup BS,E;\varpi^{\prime }:E\to Z^{\geq 0}\right)$ from *G*, where $\varpi^{\prime }\left(v_{i},v_{j}\right)=\xi_{1}\cdot \frac{h\left(v_{i}\right)+h\left(v_{j}\right)}{2}+\xi_{2}\cdot f\left(v_{i},v_{j}\right)$;2:Sort the *n* PoIs $v_{1},v_{2},\ldots ,v_{n}$ in *V* by their maximum data collection deadlines in non-decreasing order, i.e., $T_{1}\leq T_{2}\leq \cdots \leq T_{n}$, where n=|V|;3:For each PoI $v_{i}$, let $T_{i}^{\prime }=2^{\left\lfloor \log_{2}\left(T_{i}/T_{1}\right)\right\rfloor }\cdot T_{1}$;4:Divide the set *V* into *p* subsets $V_{0}$, $V_{1}$, ⋯, $V_{p}$, where PoI $v_{i}$ is contained in $V_{q}$ if $2^{q}T_{1}=2^{\left\lfloor \log_{2}\left(T_{i}/T_{1}\right)\right\rfloor }T_{1}$, 0≤q≤p, and $p=\lfloor \log_{2}\left(T_{n}/T_{1}\right)\rfloor$. By the definition of each set $V_{q}$, the PoIs in $V_{q}$ have the same data collection delay $2^{q}T_{1}$;5:**for** *q*←1 to *p* **do**6: Obtain a subgraph $G_{q}^{\prime }\left[BS\cup V_{0}\cup V_{1}\cup \cdots \cup V_{q}\right]$ of the graph $G^{\prime }$;7: For the subgraph $G_{q}^{\prime }\left[BS\cup V_{0}\cup V_{1}\cup \cdots \cup V_{q}\right]$, construct a set $D_{q}=\left\{D_{q,1},D_{q,2},\ldots ,D_{q,K}\right\}$ of *K* flying tours by invoking the algorithm for the vehicle routing problem with the objective of minimizing the total cost [19];8:**end for**9:C←0;10:**for** *j*←1 to 2*^p^* do11: Let $C_{j}=D_{q}$, where *q* is the largest integer in range $\left[0,\left\lfloor \log_{2}j\right\rfloor \right]$ so that $j\;\mathrm{mod}\;2^{q}=0$;12: $C\leftarrow C\cup \{\left(C_{j},\left(j-1\right)\cdot T_{1}\right)\}$;13:**end for**14:**for** *m*′←2 to ⌊*T*/*T*′_*n*_⌋ **do**15: **for** *j*←1 to 2^*p*^ **do**16:  $C=C\cup \{C_{j},\left(m^{\prime }-1\right)\cdot T_{n}^{\prime }+\left(j-1\right)\cdot T_{1}\}$**return**C.17: **end for**18:**end for**19:**return** C

**Figure 4:**
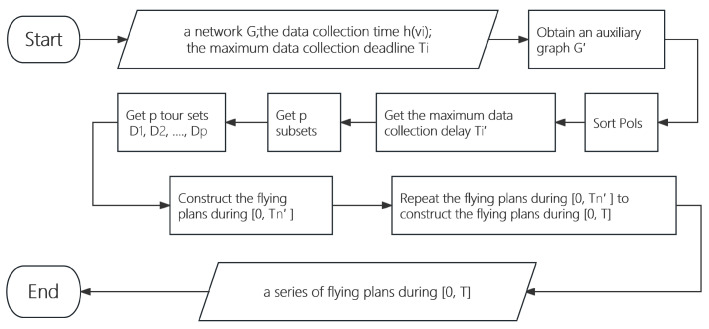
Algorithm flowchart of Algorithm 1.

Figure 4 shows the algorithm flowchart of Algorithm 1.

### 3.3. Algorithm Analysis

The main focus in this subsection is to prove the approximation ratio as well as the time complexity of the proposed algorithm.

In the following, it firstly shows that the constructed auxiliary graph $G^{\prime }$ is a metric graph since the cited algorithm for the vehicle routing problem with the objective of minimizing the total cost [19] can only be used in a metric graph.

**Lemma** **2.** *The constructed auxiliary graph $G^{\prime }$ is a metric graph.*

**Proof.** ** ** In the auxiliary graph $G^{\prime }=\left(V\cup BS,E^{\prime };\varpi^{\prime }:E^{\prime }\to Z^{\geq 0}\right)$, there is an edge between any two nodes in the set V∪BS. In the following, it will be shown that the weight of any edge in the graph $G^{\prime }$ satisfy the Triangular Inequality Theorem. For any three nodes $v_{i}$, $v_{j}$, $v_{k}$ in the graph, there are

(16) $$\begin{array}{ccc} & & \varpi^{\prime }\left(v_{i},v_{j}\right)+\varpi^{\prime }\left(v_{i},v_{k}\right)\\ & = & \left(\xi_{1}\cdot \frac{h\left(v_{i}\right)+h\left(v_{j}\right)}{2}+\xi_{2}\cdot f\left(v_{i},v_{j}\right)\right)+\left(\xi_{1}\cdot \frac{h\left(v_{i}\right)+h\left(v_{k}\right)}{2}+\xi_{2}\cdot f\left(v_{i},v_{k}\right)\right)\\ & = & \xi_{1}\cdot \frac{h\left(v_{k}\right)+h\left(v_{j}\right)}{2}+\xi_{1}\cdot h\left(v_{i}\right)+\xi_{2}\cdot f\left(v_{i},v_{j}\right)+\xi_{2}\cdot f\left(v_{i},v_{k}\right)\\ & \geq & \xi_{1}\cdot \frac{h\left(v_{k}\right)+h\left(v_{j}\right)}{2}+\xi_{2}\cdot f\left(v_{i},v_{j}\right)+\xi_{2}\cdot f\left(v_{i},v_{k}\right)\\ & \geq & \xi_{1}\cdot \frac{h\left(v_{k}\right)+h\left(v_{j}\right)}{2}+\xi_{2}\cdot f\left(v_{k},v_{j}\right)\\ & = & \varpi^{\prime }\left(v_{k},v_{j}\right). \end{array}$$

This proves that the auxiliary graph $G^{\prime }$ is a metric graph.    □

**Lemma** **3.** Given a set V of PoIs and a data update deadline $T_{i}$ for each PoI $v_{i}$ in V, divide the set V into p+1 sub-sets of PoIs $V_{0}$, $V_{1}$, …, $V_{p}$, where the PoIs in each set $V_{q}\;\left(0\leq q\leq p\right)$ is assigned a same data collection delay $2^{q}T_{1}$. Let OPT be the optimal value of the UAV service cost minimization problem in the graph $G\left[BS\cup V_{0}\cup V_{1}\cup \cdots \cup V_{p}\right]=G\left[BS\cup V\right]$ (i.e., the graph G). Then, OPT is also the optimal value of the UAV service cost minimization problem in the graph $G^{\prime }\left[BS\cup V\right]$ (i.e., the graph $G^{\prime }$). In the sub-graph $G_{q}^{\prime }\left[BS\cup V_{0}\cup V_{1}\cup \cdots \cup V_{q}\right]$ of the graph $G^{\prime }\left[BS\cup V\right]$, let $D_{q}^{*}=\left\{D_{q,1},D_{q,2},\ldots ,D_{q,K}\right\}$ be an optimal solution to the vehicle routing problem with the objective of minimizing the total cost [19]. Then, there is $\varpi^{\prime }\left(D_{q}^{*}\right)\leq \frac{OPT}{m\cdot 2^{p-q}}$, where $\varpi^{\prime }\left(D_{q}^{*}\right)=\sum_{l=1}^{K}\varpi^{\prime }\left(D_{q,l}^{*}\right)$, 0≤q≤p and $p=\lfloor \log_{2}\left(T_{n}/T_{1}\right)\rfloor$.

**Proof.** ** ** In order to prove $\varpi^{\prime }\left(D_{q}^{*}\right)\leq \frac{OPT}{m\cdot 2^{p-q}}$, some intermediate variables are introduced to aid the proof. Here, we first divide the monitoring period *T* of the entire network, i.e., $2mT_{n}^{\prime }$$\left(=2m\cdot 2^{p}T_{1}\right)$, into $m\cdot 2^{p-q}$ time slices on average, where the length of each time slice is $\left(2m\cdot 2^{p}T_{1}\right)/\left(m\cdot 2^{p-q}\right)=2^{q+1}T_{1}$. The monitoring period *T* is then divided into $\left(0,2^{q+1}T_{1}\right]$, $\left(2^{q+1}T_{1},2\cdot 2^{q+1}T_{1}\right]$, *…*, $\left(\left(j-1\right)\cdot 2^{q+1}T_{1},j\cdot 2^{q+1}T_{1}\right]$, *…*, $\left(\left(m\cdot 2^{p-q}-1\right)\cdot 2^{q+1}T_{1},\left(m\cdot 2^{p-q}\right)\cdot 2^{q+1}T_{1}\right]$. And the range of the *j*-th ($1\leq j\leq m\cdot 2^{p-q}$) time slice is $\left(\left(j-1\right)\cdot 2^{q+1}T_{1},j\cdot 2^{q+1}T_{1}\right]$.

Suppose that the optimal solution to the UAV service cost minimization problem in graph $G^{\prime }\left[BS\cup V\right]$ is a composition of *l* optimal data collection plans starting from the moments $t_{1}^{*}$, $t_{2}^{*}$, *…*, $t_{l}^{*}$, respectively, which is denoted as $\left(C_{1}^{*},t_{1}^{*}\right)$, $\left(C_{2}^{*},t_{2}^{*}\right)$, …, $\left(C_{l}^{*},t_{l}^{*}\right)$, where $0\leq t_{1}^{*}\leq t_{2}^{*}\leq \ldots \leq t_{l}^{*}<T$, $C_{s}^{*}=\left\{C_{s,1}^{*},C_{s,2}^{*},\ldots ,C_{s,K}^{*}\right\}$, and 1≤s≤l. Recall that OPT is the optimal value of the UAV service cost minimization problem in graph $G^{\prime }\left[BS\cup V\right]$, i.e., $OPT=\sum_{s=1}^{l}\varpi^{\prime }\left(C_{s}^{*}\right)=\sum_{s=1}^{l}\sum_{k=1}^{K}\varpi^{\prime }\left(C_{s,k}^{*}\right)$, where *K* is the number of UAVs. According to $m\cdot 2^{p-q}$ time slices $\left(0,2^{q+1}T_{1}\right]$, $\left(2^{q+1}T_{1},2\cdot 2^{q+1}T_{1}\right]$, *…*, $\left(\left(j-1\right)\cdot 2^{q+1}T_{1},j\cdot 2^{q+1}T_{1}\right]$, *…*, $\left(\left(m\cdot 2^{p-q}-1\right)\cdot 2^{q+1}T_{1},\left(m\cdot 2^{p-q}\right)\cdot 2^{q+1}T_{1}\right]$, and the start time $t_{s}^{*}$ in each optimal data collection plan $\left(C_{s}^{*},t_{s}^{*}\right)$ in the optimal solution, we divide the *l* optimal data collection plans into $m\cdot 2^{p-q}$ disjoint groups. The specific division rule is that if the start time $t_{s}^{*}$ of the *s*-th optimal data collection plan $\left(C_{s}^{*},t_{s}^{*}\right)$ is in the *j*-th time slice, i.e., $\left(j-1\right)\cdot 2^{q+1}T_{1}<t_{s}^{*}\leq j\cdot 2^{q+1}T_{1}$, then $\left(C_{s}^{*},t_{s}^{*}\right)$ is classified into the *j*-th group, where 1≤s≤l and $1\leq j\leq m\cdot 2^{p-q}$. Let $\psi_{j}$ be the the *j*-th group of the optimal data collection plans and $\varpi^{\prime }\left(\psi_{j}\right)$ be the consumed energy of the UAVs in the *j*-th group, i.e., $\varpi^{\prime }\left(\psi_{j}\right)=\sum_{C_{s}^{*}\in \psi_{j}}\varpi^{\prime }\left(C_{s}^{*}\right)$.

In order to prove that $\varpi^{\prime }\left(D_{q}^{*}\right)\leq \frac{OPT}{m\cdot 2^{p-q}}$, we later prove the following two points for the $m\cdot 2^{p-q}$ time slices of length $2^{q+1}T_{1}$:

(i) There is at least one time slice *j* such that the UAV consumed energy $\varpi^{\prime }\left(\psi_{j}\right)$ in the *j*-th optimal data collection plan group $\psi_{j}$ partitioned in that time slice is not greater than $m\cdot 2^{p-q}$ percent of the optimal solution OPT, i.e., $\varpi^{\prime }\left(\psi_{j}\right)\leq \frac{OPT}{m\cdot 2^{p-q}}$.
(ii) Using the *j*-th group $\psi_{j}$ of optimal data collection plans corresponding to the *j*-th time slice, we can construct a feasible solution $C_{q}^{f}$ to the vehicle routing problem with the objective of minimizing the total cost in graph $G_{q}^{\prime }\left[BS\cup V_{0}\cup V_{1}\cup \cdots \cup V_{q}\right]$, where the consumed energy $\varpi^{\prime }\left(C_{q}^{f}\right)$ is not greater than the consumed energy $\varpi^{\prime }\left(\psi_{j}\right)$, i.e., $\varpi^{\prime }\left(C_{q}^{f}\right)\leq \varpi^{\prime }\left(\psi_{j}\right)$.

Since $D_{q}^{*}$ is an optimal solution to the vehicle routing problem with the objective of minimizing the total cost in the graph $G_{q}^{\prime }\left[BS\cup V_{0}\cup V_{1}\cup \cdots \cup V_{q}\right]$, and $C_{q}^{f}$ is a feasible solution to the same problem in the same graph, thus there is $\varpi^{\prime }\left(D_{q}^{*}\right)\leq \varpi^{\prime }\left(C_{q}^{f}\right)$. If the conclusions in (i) and (ii) above hold, it is obvious that $\varpi^{\prime }\left(D_{q}^{*}\right)\leq \varpi^{\prime }\left(C_{q}^{f}\right)\leq \varpi^{\prime }\left(\psi_{j}\right)\leq \frac{OPT}{m\cdot 2^{p-q}}$.

First, the proof of (i) is given in the following, using the reduction to absurdity. In the $m\cdot 2^{p-q}$ time slices $\left(0,2^{q+1}T_{1}\right]$, $\left(2^{q+1}T_{1},2\cdot 2^{q+1}T_{1}\right]$, *…*, $\left(j-1\right)\cdot 2^{q+1}T_{1},j\cdot 2^{q+1}T_{1}]$, *…*, $\left(\left(m\cdot 2^{p-q}-1\right)\cdot 2^{q+1}T_{1},\left(m\cdot 2^{p-q}\right)\cdot 2^{q+1}T_{1}\right]$, we first assumed that there does not exist a time slice $j\;(1\leq j\leq m\cdot 2^{p-q})$ of length $2^{q+1}T_{1}$ such that the consumed energy of the UAVs in that time slice is not greater than $\left(m\cdot 2^{p-q}\right)$ percent of the optimal solution OPT. Under the assumption, we have that the UAV consumed energy in all time slices of length $2^{q+1}T_{1}$ is greater than $\left(m\cdot 2^{p-q}\right)$ percent of the optimal value OPT. Thus, we have that the total UAV consumed energy in the $m\cdot 2^{p-q}$ time slices is equal to $\sum_{j=1}^{m\cdot 2^{p-q}}\varpi^{\prime }\left(\psi_{j}\right)>m\cdot 2^{p-q}\cdot \frac{OPT}{m\cdot 2^{p-q}}=OPT$. But, according to the definition of $\psi_{j}$, there is $\sum_{j=1}^{m\cdot 2^{p-q}}\varpi^{\prime }\left(\psi_{j}\right)=OPT$, which contradicts the conclusion obtained above. Therefore, the assumption is not valid. And among the $m\cdot 2^{p-q}$ data collection plans, there must exist a certain group $\psi_{j}$ of optimal data collection plans such that the UAV consumed energy $\varpi^{\prime }\left(\psi_{j}\right)$ in that group is no more than $\left(m\cdot 2^{p-q}\right)$ percent of the optimal value OPT, i.e.,

(17) $$\varpi^{\prime }\left(\psi_{j}\right)\leq \frac{OPT}{m\cdot 2^{p-q}}.$$

Using the *j*-th group of data collection plan $\psi_{j}$ corresponding to the *j*-th time slice, we can construct a feasible solution $C_{q}^{f}$ to the vehicle routing problem with the objective of minimizing the total cost in graph $G_{q}^{\prime }\left[BS\cup V_{0}\cup V_{1}\cup \cdots \cup V_{q}\right]$, where the consumed energy $\varpi^{\prime }\left(C_{q}^{f}\right)$ is not greater than the consumed energy $\varpi^{\prime }\left(\psi_{j}\right)$, i.e., $\varpi^{\prime }\left(C_{q}^{f}\right)\leq \varpi^{\prime }\left(\psi_{j}\right)$.

Then, we prove (ii), the UAV consumed energy $\varpi^{\prime }\left(\psi_{j}\right)$ in the *j*-th group $\psi_{j}$ of optimal data collection plans is not less than the UAV consumed energy $\varpi^{\prime }\left(C_{q}^{f}\right)$ of a feasible solution $C_{q}^{f}$ for the vehicle routing problem with the objective of minimizing the total cost in graph $G_{q}^{\prime }\left[BS\cup V_{0}\cup V_{1}\cup \cdots \cup V_{q}\right]$. On the basis of the *j*-th group $\psi_{j}$ of optimal data collection plans, we construct a feasible solution $C_{q}^{f}=\left\{C_{q,1}^{f},C_{q,2}^{f},\ldots ,C_{q,K}^{f}\right\}$ for the vehicle routing problem with the objective of minimizing the total cost in graph $G_{q}^{\prime }\left[BS\cup V_{0}\cup V_{1}\cup \cdots \cup V_{q}\right]$ and such that the UAV consumed energy of $C_{q}^{f}$ is not greater than the UAV consumed energy $\varpi^{\prime }\left(\psi_{j}\right)$ in the *j*-th group $\psi_{j}$ of optimal data collection plans.

For all optimal flying tours contained in the set $\psi_{j}$, since all tours connect to the base station BS, then these tours form a connected graph. In this connected graph, the degree of the base station BS is 2K and the degrees of other PoIs are 2; i.e., the degrees of all nodes in the connected graph are even numbers. Thus, these tours form a Euler graph. Since there must be a Euler circuit in a Euler graph, all the flying tours contained in the set $\psi_{j}$ are also a Euler circuit, which is denoted as $C_{j}^{o}$, and $\varpi^{\prime }\left(C_{j}^{o}\right)=\varpi^{\prime }\left(\psi_{j}\right)$.

Here, we first show that in the *j*-th data collection plan set $\psi_{j}$ corresponding to the *j*-th time slice, all PoIs in $\cup_{i=0}^{q}V_{i}$ are visited at least once. Suppose that there is a PoI $v_{i}$ in $\cup_{i=0}^{q}V_{i}$ which is not involved in the data collection plan set $\psi_{j}$. Since $v_{i}\in \cup_{i=0}^{q}V_{i}$, combined with Ineq. (14), for the data update duration $T_{i}$, we have $T_{i}<2\cdot T_{i}^{\prime }\leq 2\cdot 2^{q}T_{1}=2^{q+1}T_{1}$. Since we suppose that $v_{i}$ is not involved in the data collection plan set $\psi_{j}$, its data update duration $T_{i}$ must be greater than the length of the time slice $2^{q+1}T_{1}$, i.e., $T_{i}>2^{q+1}T_{1}$. Clearly, the description in the above sentence is contradictory, so the assumption is not valid and $v_{i}$ must have been visited once in some of the data collection plan set $\psi_{j}$.

As we show in the previous paragraph, in the *j*-th set $\psi_{j}$ of optimal data collection plans, all PoIs in $\cup_{i=0}^{q}V_{i}$ are visited at least once. By removing recurring edges and the PoIs that are not contained in $\cup_{i=0}^{q}V_{i}$ from the Euler circuit $C_{j}^{o}$, we obtain a tour $C_{q}^{\prime }$ that contains only the PoIs in $\cup_{i=0}^{q}V_{i}$ and the base station BS. Since the weights of the edges all follow the Triangular Inequality Theorem, we have

(18) $$\varpi^{\prime }\left(C_{q}^{\prime }\right)\leq \varpi^{\prime }\left(C_{j}^{o}\right)=\varpi^{\prime }\left(\psi_{j}\right).$$

As it has been proved in the *j*-th data collection plan set $\psi_{j}$ corresponding to the *j*-th time slice, all PoIs in $\cup_{i=0}^{q}V_{i}$ are visited at least once. Then, we have $\cup_{i=0}^{q}V_{i}\subseteq V\left(C_{q}^{\prime }\right)$. Since the degree of the base station BS in the tour $C_{q}^{\prime }$ is 2K, the tour $C_{q}^{\prime }$ can be partitioned into *K* sub-tour $C_{q,1}^{f}$, $C_{q,2}^{f}$, *…*, and $C_{q,K}^{f}$ that each sub-tour contains BS. Let $C_{q}^{f}$ be the set of tours $C_{q,1}^{f}$, $C_{q,2}^{f}$, *…*, $C_{q,K}^{f}$, i.e., $C_{q}^{f}=\left\{C_{q,1}^{f},C_{q,2}^{f},\ldots ,C_{q,K}^{f}\right\}$. Obviously, $C_{q}^{f}$ is a feasible solution to the vehicle routing problem with the objective of minimizing the total cost in graph $G_{q}^{\prime }\left[BS\cup V_{0}\cup V_{1}\cup \cdots \cup V_{q}\right]$.

According to Ineq. (18), we have $\varpi^{\prime }\left(C_{q}^{f}\right)=\varpi^{\prime }\left(C_{q}^{\prime }\right)\leq \varpi^{\prime }\left(\psi_{j}\right)$. (ii) is proved.

In the following, $\varpi^{\prime }\left(D_{q}^{*}\right)\leq \frac{OPT}{m\cdot 2^{p-q}}$ will be proved on the basis of the already proved (i) and (ii).

As $D_{q}^{*}=\left\{D_{q,1},D_{q,2},\ldots ,D_{q,K}\right\}$ is an optimal solution to the vehicle routing problem with the objective of minimizing the total cost in the graph $G_{q}^{\prime }\left[BS\cup V_{0}\cup V_{1}\cup \cdots \cup V_{q}\right]$, we have

(19) $$\varpi^{\prime }\left(D_{q}^{*}\right)=\sum_{k=1}^{K}\varpi^{\prime }\left(D_{q,k}^{*}\right)\leq \sum_{k=1}^{K}\varpi^{\prime }\left(C_{q,k}\right).$$

Combining Ineq. (17), Ineq. (18), and Ineq. (19), we have:

(20) $$\varpi^{\prime }\left(D_{q}^{*}\right)=\sum_{k=1}^{K}\varpi^{\prime }\left(D_{q,k}^{*}\right)\leq \sum_{k=1}^{K}\varpi^{\prime }\left(C_{q,k}\right)==\varpi^{\prime }\left(\psi_{j}\right)\leq \frac{OPT}{m\cdot 2^{p-q}}.$$

In summary, for the optimal solution $D_{q}^{*}$ for the vehicle routing problem with the objective of minimizing the total cost in the graph $G_{q}^{\prime }\left[BS\cup V_{0}\cup V_{1}\cup \cdots \cup V_{q}\right]$, we obtain that $\varpi^{\prime }\left(D_{q}^{*}\right)\leq \frac{OPT}{m\cdot 2^{p-q}}$, where OPT is the UAV consumed energy in the optimal solution to the UAV service cost minimization problem, 0≤q≤p, and $p=\lfloor \log_{2}\left(T_{n}/T_{1}\right)\rfloor$.    □

**Lemma** **4.** *Given a set V of PoIs and a base stations BS, the Algorithm 1 deduces a solution with an approximation ratio of 2p+4, where $p=\lfloor \log_{2}\left(T_{n}/T_{1}\right)\rfloor$, $T_{n}$ and $T_{1}$ are the maximum and minimum data update deadlines, respectively, for the PoIs in the set V.*

**Proof.** ** ** In the following, we show that the approximation ratio of the Algorithm 1 is 2p+4.

Recall that the $2m\cdot 2^{p}$ data collection plans obtained in Algorithm 1 are:

$\left(C_{1},0\right)$, $\left(C_{2},T_{1}\right)$, *…*, $\left(C_{2^{p}-1},\left(2^{p}-2\right)T_{1}\right)$, $\left(C_{2^{p}},\left(2^{p}-1\right)T_{1}\right)$ during time slice $T_{n}^{\prime }$;

$\left(C_{1},T_{n}^{\prime }\right)$,$\left(C_{2},T_{n}^{\prime }+T_{1}\right)$,…, $\left(C_{2^{p}-1},T_{n}^{\prime }+\left(2^{p}-2\right)T_{1}\right)$,$\left(C_{2^{p}},T_{n}^{\prime }+\left(2^{p}-1\right)T_{1}\right)$ during time slice $2T_{n}^{\prime }$;

*…*;

$\left(C_{1},\left(2m-1\right)T_{n}^{\prime }\right)$,$\left(C_{2},\left(2m-1\right)T_{n}^{\prime }+T_{1}\right)$,*…*, $\left(C_{2^{p}-1},\left(2m-1\right)T_{n}^{\prime }+\left(2^{p}-2\right)T_{1}\right)$,$\left(C_{2^{p}},\left(2m-1\right)T_{n}^{\prime }+\left(2^{p}-1\right)T_{1}\right)$ during time slice $\left(2m-1\right)T_{n}^{\prime }$;

Thus, the total UAV consumed energy in the solution obtained by Algorithm 1 over the monitoring period *T* is:

(21) $$2m\sum_{j=1}^{2^{p}}\varpi^{\prime }\left(C_{j}\right).$$

Let $T_{n}^{\prime }=\left\{\left(C_{1},0\right),\left(C_{2},T_{1}\right),\ldots ,\left(C_{2^{p}},\left(2^{p}-1\right)\cdot T_{1}\right)\right\}$ be the set of data collection plans during the period $\left[0,T_{n}^{\prime }\right]$. From the construction process of $T_{n}^{\prime }$ in the Algorithm 1, it can be seen that there are only $2^{p-q-1}$ data collection plans in $T_{n}^{\prime }$, and it only contains the PoIs in set $BS\cup V_{0}\cup V_{1}\cup \cdots \cup V_{q}$, where 0≤q≤p. For the set $BS\cup V_{0}\cup V_{1}\cup \cdots \cup V_{p}$, i.e., the set BS∪V, there is only one data collection plan in $T_{n}^{\prime }$ that contains all PoIs in BS∪V. Denote $D_{p}$ as the set of flying tours which are obtained as an approximate solution to the vehicle routing problem with the objective of minimizing the total cost in the subgraph $G_{q}^{\prime }\left[BS\cup V_{0}\cup V_{1}\cup \cdots \cup V_{p}\right]$, i.e., $G_{q}^{\prime }\left[BS\cup V\right]$, by invoking [19]. Denote the consumed energy of $D_{p}$ as $\varpi^{\prime }\left(D_{p}\right)$. Combined with Ineq. (21), there is:

(22) $$2m\sum_{j=1}^{2^{p}}\varpi^{\prime }\left(C_{j}\right)=2m\cdot \left(\varpi^{\prime }\left(D_{p}\right)+\sum_{q=0}^{p-1}2^{p-q-1}\varpi^{\prime }\left(D_{q}\right)\right).$$

In the graph $G_{q}^{\prime }\left[BS\cup V_{0}\cup V_{1}\cup \cdots \cup V_{q}\right]$(0≤q≤p), the tour set $D_{q}=\{D_{q,1},D_{q,2},\ldots ,$$D_{q,K}\}$ is obtained via the algorithm with an approximation ratio of 2 for the vehicle routing problem with the objective of minimizing the total cost [19]. Denote $\varpi^{\prime }\left(D_{q}\right)$ as the UAV consumed energy of flying tour set $D_{q}$. In the graph $G_{q}^{\prime }\left[BS\cup V_{0}\cup V_{1}\cup \cdots \cup V_{q}\right]$(0≤q≤p), let $D_{q}^{*}=\left\{D_{q,1}^{*},D_{q,2}^{*},\ldots ,D_{q,K}^{*}\right\}$ be an optimal solution to the vehicle routing problem with the objective of minimizing the total cost [19]. And denote $\varpi^{\prime }{\left(D_{q}\right)}^{*}$ as the UAV consumed energy of tours in set $D_{q}^{*}$. According to [19], we have $\varpi^{\prime }\left(D_{q}\right)\leq 2\varpi^{\prime }\left(D_{q}^{*}\right)$, where 0≤q≤p. Combing with Theorem 3 and Ineq. (20), we can obtain

(23) $$\begin{array}{ccc} 2m\sum_{j=1}^{2^{p}}\varpi^{\prime }\left(C_{j}\right) & = & 2m\cdot (\varpi^{\prime }\left(D_{p}\right)+\sum_{q=0}^{p-1}2^{p-q-1}\varpi^{\prime }\left(D_{q}\right))\\ & < & 2m\cdot (2\varpi^{\prime }\left(D_{p}^{*}\right)+2\sum_{q=0}^{p-1}2^{p-q-1}\varpi^{\prime }\left(D_{q}^{*}\right))\\ & < & 4m\cdot (\frac{OPT}{m}+\sum_{q=0}^{p-1}2^{p-q-1}\frac{OPT}{m\cdot 2^{p-q}})\\ & = & (2p+4)OPT \end{array}$$

As proved above, the approximation ratio of the Algorithm 1 is 2p+4, where $p=\lfloor \log_{2}\left(T_{n}/T_{1}\right)\rfloor$, and $T_{n}$ and $T_{1}$ are the maximum and minimum data update deadlines of PoIs in *V*, respectively.

The time complexity of Algorithm 1 is analyzed as follows. In the first step of the algorithm, the time complexity of the construction of the auxiliary graph $G^{\prime }$ is $O(n^{2})$, where *n* is the number of PoIs in the set *V*. In the second step, the time complexity of the grouping operation of PoIs in *V* is O(n). In the third step, Algorithm 1 firstly finds the data collection plans within the maximum data collection delay $T_{n}^{\prime }$, and then repeats the data collection plans within $T_{n}^{\prime }$ to obtain the data collection plans within the network monitoring period *T*. The algorithm finds *p* sets of tours in subgraphs $G^{\prime }\left[BS\cup V_{0}\right]$, $G_{1}^{\prime }\left[BS\cup V_{0}\cup V_{1}\right]$, *…*, and $G_{p}^{\prime }\left[BS\cup V_{0}\cup V_{1}\cup \cdots \cup V_{p}\right]$, respectively, through invoking the algorithm [19] with a time complexity of $O(n^{2})$. And the time complexity of the third step is $O(p\cdot n^{2})$. In summary, the time complexity of the Algorithm 1 is $O\left(n^{2}+n+p\cdot n^{2}\right)=O\left(p\cdot n^{2}\right)$, where $p=\lfloor \log_{2}\left(T_{n}/T_{1}\right)\rfloor$, $T_{n}$ and $T_{1}$ are the maximum and minimum data update deadlines, respectively, and *n* is the number of PoIs in *V*.    □

## 4. Performance Evaluation

In this section, we evaluate the performance of the proposed algorithm through extensive experiments.

### 4.1. Simulation Environment

The experimental area is set up as a 10 km × 10 km × 100 m three-dimensional space. There are 50 to 200 PoIs randomly distributed in the experimental area, and the base station BS is randomly scattered at the edges of the experimental area. The hovering time of each PoI is a number that is randomly selected from the interval $\left[h_{min},h_{max}\right]$, where the minimum hovering time $h_{min}=$ 10 s, and the maximum hovering time $h_{max}=$ 3 min = 180 s [8,10]. The number of UAVs is 10, and each UAV is located at the base station initially. The speed of UAVs s= 8 m/s [20]. The energy consumption rates of the UAVs while hovering and flying are 150 J/s and 100 J/s, respectively [17]. The monitoring period is 48 h. Each value in figures is the average of the results by applying each mentioned algorithm to 100 different network topologies with the same network size.

In order to evaluate the performance of the proposed algorithm approAlg, three existing comparison algorithms are considered. Each of the comparison algorithms is described as follows.

(1) In algorithm periodicAlg, the base station periodically dispatches UAVs to collect data from all PoIs and guarantee that the collection delays of all PoIs do not exceed the minimum data update deadline $T_{min}$. The flying tours of the UAVs is obtained by the algorithm with an approximation ratio of 2 in [19] for the vehicle routing problem with the objective of minimizing the total cost.
(2) In algorithm conAlg, the base station calculates the data collection delay for each PoI at the current moment. If the data collection delay has exceeded a given threshold (here the value is 50% of the data update deadline for the PoI), then these PoIs will be added to the set of PoIs to be visited in the next round. UAVs are dispatched to collect the data of PoIs in the set, where the flying tours of UAVs are obtained by the algorithm [19].

### 4.2. Algorithm Performance

In the following, we study the impact of the network size *n*, the maximum hovering time $h_{max}$, and the maximum data update deadline $T_{max}$.

**Figure 5:**
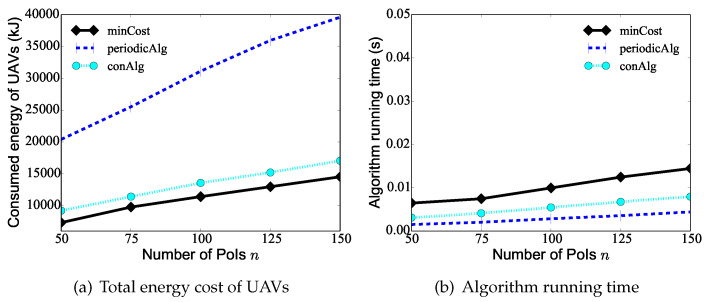
The performance of different algorithms by varying the number *n* of PoIs from 25 to 150.

We first evaluate the performance of the proposed algorithm minCost against existing algorithms periodicAlg and conAlg by varying the number *n* of PoIs from 25 to 150, while the number *K* of UAVs is 10, the data update deadline $T_{i}$ of each PoI $v_{i}$ is randomly selected from $[T_{min}=$ 20 min, $T_{max}=$ 120 min], and the maximum hovering time $h_{max}$ of PoIs is 60 s. From Figure 5a, it can be seen that as the number of PoIs increases, the UAVs need to take on a heavier monitoring task, and the total consumed energy of the UAVs increases. The figure shows the total consumed energy of the UAVs deduced by algorithm minCost is only 35% to 85% of that deduced by the two compared algorithms. For example, the total consumed energy of the UAVs by algorithms minCost, periodicAlg, and conAlg is 14,520 J, 39,600 J, 17,040 J, respectively, when there are n=150 PoIs in the area. Figure 5b shows the running time of algorithm minCost, algorithm periodicAlg, and algorithm conAlg. In the following, we do not compare the running times of the algorithms since the curves are similar.

**Figure 6:**
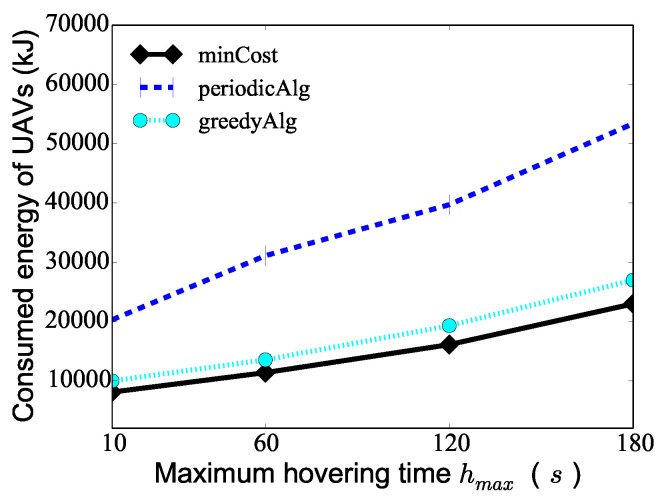
The performance of different algorithms varying the maximum hovering time $h_{max}$ of PoIs.

We then investigate the algorithm performance varying the maximum hovering time $h_{max}$ from 10 s to 180 s when the number *K* of UAVs is 10, $T_{max}=120$ min, and the number of PoIs 100. Figure 6 demonstrates that as the maximum hovering time $h_{max}$ increases, the consumed energy of the UAVs deduced by algorithm minCost, algorithm periodicAlg, and algorithm conAlg increases. The reason for this is that as the maximum hovering time increases, the UAVs consume more energy on hovering, which leads to the increase in the total consumed energy of the UAVs. The figure shows the total consumed energy of the UAVs deduced by algorithm minCost is only 40% to 83% of that deduced by the two compared algorithms. For example, the total consumed energy of the UAVs by algorithms minCost, periodicAlg, and conAlg is 16,120 J, 39,720 J, and 19,320 J, respectively, when the maximum hovering time $h_{max}=120$ s.

**Figure 7:**
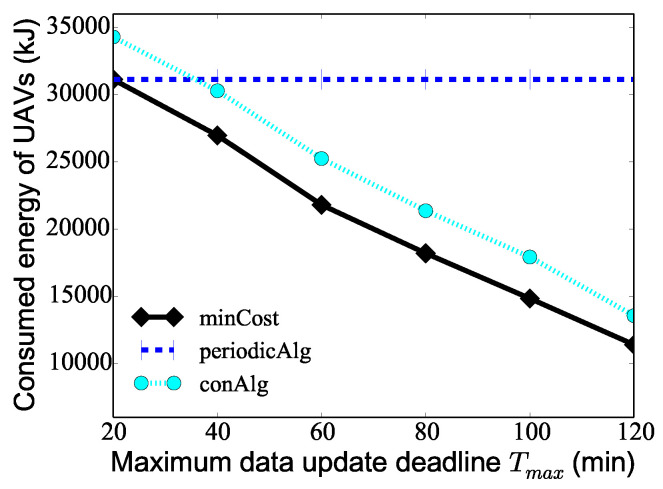
The performance of different algorithms varying the maximum data collection deadline $T_{max}$ of PoIs.

We finally investigate the algorithm performance by varying the maximum data update deadline $T_{max}$ from 20 min to 120 min when the number *K* of UAVs is 10, the number of PoIs 100, and the maximum hovering time $h_{max}$ of PoIs is 60s. Figure 7 demonstrates that as the maximum data update deadline $T_{max}$ increases, the consumed energy of the UAVs deduced by both algorithm minCost and algorithm conAlg decreases. The reason for this is that as the maximum data update deadline increases, the number of times that PoIs are visited in the whole monitoring period *T* deduced by algorithm minCost and algorithm conAlg decreases, so the total consumed energy of the UAVs obtained by the two algorithms decreases. For the algorithm periodicAlg, since the algorithm only cares about the minimum monitoring period $T_{min}$ of the PoIs, the change of $T_{max}$ has no effect on the result of the algorithm, and the graph of algorithm periodicAlg shows a flat straight line. It is worth noting that when $T_{max}=T_{min}=20$ min, the algorithm minCost also degenerates into the algorithm periodicAlg, and therefore, the results of the two algorithms are the same. From the figure, it can be seen that the consumed energy of the UAVs is effectively reduced when considering the different data collection deadlines of the PoIs.

## 5. Conclusions

In this paper, we consider a scenario in which PoIs are visited multiple times during a long monitoring period *T* to persistently update the data of the PoIs. And we also take into account the data collection deadlines of different PoIs as the importance of PoIs varies. In the above scenario, a UAV service cost minimization problem is proposed, which aims to find a series of data collection plans for a given number of UAVs over the monitoring period *T*, such that the service cost (the total consumed energy) of the UAVs is minimized over *T*. For the UAV service cost minimization problem, a new algorithm is proposed and the approximation ratio of the proposed algorithm is 2p+4, where $p=\lfloor \log_{2}\left(T_{n}/T_{1}\right)\rfloor$, and $T_{n}$ and $T_{1}$ are the maximum and minimum data update deadlines of the PoIs in *V*, respectively. The main idea of the proposed algorithm is to divide the PoIs into groups, collect the data of the PoIs according to the groups, and then deal with the UAV service cost minimization problem by invoking the algorithm for the vehicle routing problem with the objective of minimizing the total cost [19]. The performance of the proposed algorithm is also verified by a series of experiments, and the experimental results show that the total consumed energy obtained by the proposed algorithm is only 35% to 85% of those obtained by the comparison algorithms, which can effectively reduce the service cost of the UAVs during the monitoring period.

## Data Availability

Data are contained within the article.
